# Serological detection of brucellosis among febrile, malaria-negative children and domesticated dogs in an urban African setting

**DOI:** 10.4102/ajlm.v9i1.864

**Published:** 2020-09-30

**Authors:** John B. Kalule, Joseph Tomusange, Teddy Namatovu

**Affiliations:** 1 Department of Biotechnical and Diagnostic Sciences, College of Veterinary Medicine, Animal Resources Animal and Biosecurity, Kampala, Uganda

**Keywords:** brucellosis, serology, malaria, febrile illness, diagnostics

## Abstract

**Background:**

Childhood brucellosis and malaria are co-endemic febrile illnesses in some sub-Saharan African countries. Malaria and brucellosis co-infection or brucellosis sole infections are often missed due to an over emphasis on malaria and the lack of appropriate diagnostic infrastructure. Brucellosis in dogs is usually overlooked and yet there is extensive contact between humans and their pets.

**Objective:**

This study investigated brucellosis in children and dogs using a confirmatory serological testing series that screens for three *Brucella* sp.

**Methods:**

Residual blood samples from malaria smear-negative febrile children were collected and tested for *Brucella* sp and malaria parasite. During the same period, residual blood samples presented to a veterinary microbiology laboratory in the same area were tested for brucellosis using the same approach.

**Results:**

A total of 105 human and 80 canine blood samples were tested for brucellosis antibodies. The seroprevalence of brucellosis was 22.86% (25/105) in children and 1.3% (1/80) in dogs using the Card, buffered acidified plate antigen, and standard plate agglutination tests but was 0% using the rivanol precipitation plate agglutination test.

**Conclusion:**

Given that brucellosis can be caused by both smooth and rough colony strains, there is a need to modify the current serological surveillance strategy (targeted at only *Brucella abortus* and other smooth colony *Brucella* strains) to figure out the relative contribution of rough colony *Brucella* strains (*B. ovis* and *B. canis*). Since Uganda is endemic for brucellosis there is a need to modify the brucellosis surveillance strategy.

## Introduction

Globally, human brucellosis remains the most common zoonotic disease with more than 500 000 new cases annually, most of which are in sub-Saharan Africa.^1^ Generally, brucellosis has no age predilection; its transmission is linked to use of or contact with known sources of infection, such as consumption of poorly made dairy products,^2,3^ or contact with carcasses or aborted material of infected livestock. Childhood brucellosis has previously been reported among children presenting with pyrexia of unknown origin.^4^ Childhood brucellosis accounts for up to one-third of all cases of human brucellosis in endemic regions.^5^

Uganda is one of the sub-Saharan Africa countries that are endemic for brucellosis and have, according to World Organisation for Animal Health (OIE) data, reported cases of human brucellosis.^6^ As per a 2006 study which estimated brucellosis globally, Uganda had an estimated incidence of 1.8 new cases per 100 000 population.^7^

Both the vaccine strain (*Brucella abortus* S19) and the wild strains (*B. abortus, B. melitensis, B. canis* and *B. suis*) have been shown to be excreted in substantial amounts in livestock milk,^8,9,10^ a wholesome nutritious option for weaned children,^11^ thus milk is a vehicle of brucellosis transmission to humans.^12^ Milk and/or milk products should be subjected to various physical sterilisation to kill potential pathogens such as *Brucella* spp.^13^ However, due to various socio-economic factors, consumption of milk and milk products remains a risk factor for brucellosis in many regions in sub-Saharan Africa.^10,14,15,16,17^ Nevertheless, other risk factors, such as environmental contamination with aborted foetal material, or contact with infected domesticated dogs or their body fluids, could potentially be associated with brucellosis,^18^ even among children. Dogs have been proven to be reservoirs of highly pathogenic canine *B. suis* strains that can cause severe disease in humans^19^ and/or re-emergence of brucellosis on livestock farms.^20^

The clinical presentation of brucellosis is largely similar to that of malaria (high fever, joint pain, malaise, headache and chills)^21^; thus, there is a need to deploy sensitive, specific, rapid, and cost-effective laboratory diagnostic tools to differentiate them and avoid misdiagnosis.^3^ The situation is aggravated by the presence of the weak laboratory infrastructure in sub-Saharan Africa.^22^ Moreover, the morbidity and mortality rates associated with malaria are high, yet co-infections with brucellosis in this setting are not uncommon.^3^ Children presenting with high fevers, headaches, and joint pains are more likely to be treated empirically for malaria.^23^

The diagnosis of brucellosis requires the use of several clinical investigative techniques across haematology, biochemistry, radiology, bacteriology, serology and molecular biology.^24^ Isolation of *Brucella* spp. from the blood, bone marrow or other tissue fluids is the gold standard.^5^ Unlike most high-income countries where brucellosis affects mostly animals, in sub-Saharan Africa it is arguably endemic in both humans and animals.^25^ In North America, where brucellosis affects both domestic (such as cattle and goats) and wild animals (such as elk and bison), highly efficient serological tests have been developed as part of surveillance diagnostic test schemes for both.^26^ Seven such serological tests, the Card (Rose Bengal) test, the complement fixation test, the rivanol precipitation plate agglutination (RPPA) test, the standard plate agglutination (SP) test (SPT), the buffered acidified plate antigen (BAPA) test, the rapid automated presumptive test, and the fluorescence polarisation assay have been approved by the United States Department of Agriculture for the detection of *B. abortus* antibodies. *Brucella abortus* is the most prevalent species in North America. The rapid slide agglutination test is often used to test for *B. canis* in dogs which can also be infected with *B. suis.*^27^ The *B. abortus* serological tests that are commonly used cannot detect antibodies to the rough colony variants *B. canis* and *B. ovis*. The North American serological tests have rarely been used in the endemic African regions; in fact, only the Card test has been used frequently for serodiagnosis of brucellosis at the point of care for both humans and animals.^28^ The Card test is highly efficient for diagnosis of human brucellosis, often outperforming tests that take longer to perform such as Coombs, competitive enzyme-linked immunosorption assay, BrucellaCapt (immunocapture agglutination test; Vircell, Granada, Spain),^29^ immunochromatography, and immunoprecipitation with *Brucella* proteins.^30^ With the exception of the RPPA and the fluorescence polarisation tests, the SPT, Card, rapid automated presumptive and BAPA tests all have the limitation that immunoglobulin M antibodies against *Brucella* spp. cross-react with those of other Gram-negative bacteria such as *Salmonella* spp. On the other hand, the immunoglobulin G antibodies that are detected using the RPPA test do not cross-react and thus give fewer false positives. Unlike seropositivity by Rose Bengal test or BAPA, which in addition to indicating infection may also mean recovery from brucellosis, RPPA test seropositivity can be used to differentiate between active infection or past infection.^12^

Most studies of *Brucella* spp. seroprevalence in Uganda have used the Rose Bengal test (Card test) without using a confirmatory serological test as categorised by the OIE.^31,32,33^ The RPPA test is commonly used as a confirmatory test, because the non-specific reactivity is reduced by precipitation of high molecular weight serum glycoproteins.^12^ The increased specificity means a reduction in the number of false positives. This study aimed to establish the seroprevalence of brucellosis due to *B. abortus* and *B. suis* in humans presenting with acute febrile illness and domesticated dogs in the same urban African setting. We hypothesised that the use of a confirmatory serological test (RPPA) in addition to routinely used screening serological tests (BAPA, Card and SPT) would enable us to rule out positive reactions due to vaccination of tested animals, previous infection history, or cross reactions^34^ and thus figure out the true seroprevalence of brucellosis due to *B. abortus* and *B. suis* in this setting.

## Methods

### Ethical considerations

Ethical clearance was obtained from the Makerere University, College of Veterinary Medicine, Animal Resources and Biosecurity (CoVAB), School of Biomedical and Biotechnical Laboratory Sciences, Research and Ethics Committee (SBLS/REC/13/019). The ethical clearance was for use of residual blood samples from the paediatric ward at the clinical microbiology laboratory at Mulago National Referral Hospital. The same clearance also permitted the use of residual blood samples from dogs at a small animal veterinary clinic at Makerere University College of Veterinary Medicine Animal resources and Biosecurity. This study did not directly recruit or collect samples from either the febrile children or the febrile dogs.

Data on the ages and sex of the patients from whom the samples were collected were recorded. The samples were re-assigned a study number and all patient-identifying data were not accessed. The same was done for the residual blood samples from febrile dogs.

### Study design and residual sample selection

We opted to test residual blood samples from febrile children that were malaria smear-negative and residual blood samples from febrile dogs. The dogs, though owned by individual homes, closely interacted with the community in the study region; for instance, the pig abattoir in the community is potentially a common source of infection to both humans (via meat purchases) and dogs (either the abattoir waste was purchased by the dog owner as dog feed or the dogs accessed poorly disposed abattoir waste).

**FIGURE 1:**
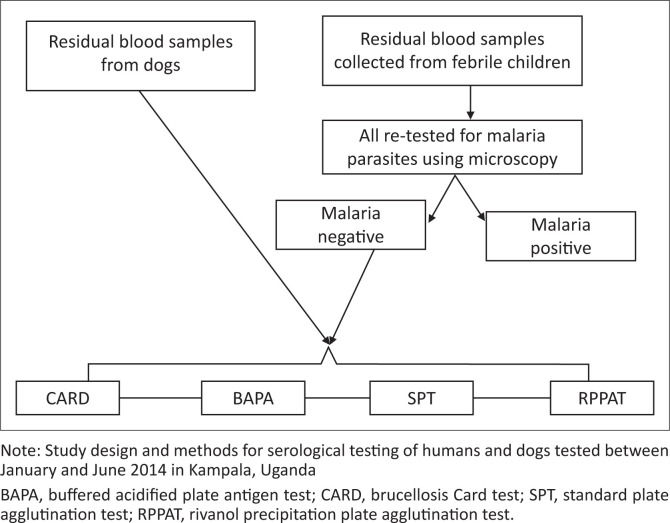
Sample testing algorithm for blood samples from children and dogs in Kampala, Uganda (January and June 2014).

All samples used were collected between January and June 2014 (Figure 1). The malaria-negative residual paediatric blood samples were collected from the clinical microbiology laboratory of a regional referral hospital, while residual canine blood samples were from a local veterinary clinic which serves the central region. The residual blood samples were tested for brucellosis using three screening tests and one confirmatory test.

#### Re-testing human blood samples for malaria

The included malaria-negative paediatric residual blood samples were re-tested for malaria to confirm that they were malaria-negative. The Field’s stains A and B (Himedia Laboratories Limited, Mumbai, India) were used to stain malaria parasites and were viewed using light microscopy. Briefly, a drop of blood was placed on a clean 25 mm x 75 mm glass slide (Merck, Darmstadt, Germany) and a thick smear was made at the centre of the glass slide using the edge of another glass slide to spread to an area of 1 cm^2^. The smear was allowed to dry at room temperature for 1 min. It was then sequentially dipped into Field’s stain A (Himedia Laboratories Limited, Mumbai, India) for 3 seconds and washed in de-ionised water for 3 s with gentle agitation, then it was dipped in Field’s stain B for 3 s and washed in tap water for 5 s. The stained smear was then air dried and examined under a light microscope at X100 magnification under oil immersion for malaria parasites, as previously described.^35^

#### Testing of human and canine sera samples for *Brucella abortus/suis* antibodies

Human blood samples that were negative for malaria were processed by centrifugation and the serum obtained was tested for brucellosis using: the Becton Dickinson Brucellosis Card Test Kit 306® (National Veterinary Services Laboratory [NVSL], Ames, Iowa, United States) – a screening test for antibodies against *B. abortus*/*B. suis* in serum and plasma; SPT (NVSL, Ames, Iowa, United States); BAPA (SL, Ames, Iowa, United States) and RPPA (NVSL, Ames, Iowa, United States) – a precipitation and agglutination test.

The BAPA, RPPA, Card and SP kits screened for *B. abortus/ B. suis* antibodies in serum and plasma; tests were carried out according to the manufacturers’ instructions on all human and canine sera included in the study. Except otherwise stated, the control sera used were the *B. abortus* complement fixation medium positive control serum (NVSL reagent 12-M) and the *B. abortus* complement fixation negative control serum (NVSL reagent 12-N).

#### Buffered acidified plate antigen test

Aseptically, 80 *μ*L of each sample or control serum was pipetted onto separate squares (1.25 inch – 1.5 inch) etched on a 12.5-inch x 12.5-inch white ceramic bioassay glass plate. Then 30 *μ*L of buffered *Brucella* plate antigen (NVSL, Ames, Iowa, United States) was added to each sample or control serum square. The serum-antigen suspension was then mixed using a glass stirrer to a diameter of approximately 27 mm and incubated for 4 minutes (±30 s) at room temperature (20 °C – 26 °C) in an enclosed space. After this incubation, it was stirred again and incubated a second time at room temperature for 4 min. The slides were then read in a Minnesota box – an illuminator with an indirect source of light, a black background, and a cover to prevent evaporation of test reagents.

#### Rivanol precipitation plate agglutination test

Aseptically, 200 *μ*L of each sample or control (positive or negative) was transferred into respective (well-labelled) tubes. Thereafter, 200 *μ*L of *Brucella* rinavol solution was transferred into each tube and mixed by shaking for 1 min. The mixture was incubated at room temperature for 5 min for precipitation. The tubes were centrifuged at 3000 revolutions per minute to pellet the precipitates. Aseptically, 80 *μ*L, 40 *μ*L, 20 *μ*L and 10 *μ*L of each sample or control supernatant were dispensed onto separate squares (1.25 inch – 1.5 inch) etched on a 12.5 inch x 12.5 inch white ceramic bioassay glass plate. Then, 30 *μ*L of *B. abortus* rivanol antigen was added. The serum-antigen mixture was stirred in circles (at least 8 circles) beginning from the 1:200 serum dilutions, then proceeding to the 1:25 dilutions of sample or control sera. The bioassay plate was then incubated at room temperature (25±2 °C) in an enclosed space for 6 min (±30 s) with rotation. The incubation with rotation was repeated for a further 6 min (±30 s). The reading was taken at the end of the second incubation under an illuminator according to the manufacturer’s instructions.

#### Card test

Aseptically, 30 *µ*L of sample or control (positive or negative) were dispensed onto the reaction area on the card. Subsequently, two drops of the *B. abortus* Card test antigen (NVSL, Ames, Iowa, United States) were then dispensed onto the card adjacent to the sample. The antigen and serum were mixed using a clean wooden stirrer for approximately 15 s, followed by rocking movements for 4 min.

#### Standard plate agglutination tests

Aseptically, 80 *µ*L, 40 *µ*L, 20 *µ*L and 10 *µ*L of sample or control serum were dispensed onto separate squares on a white ceramic bioassay glass plate. Following gentle mixing, 30 *µ*L of antigen was dispensed to each square containing sample or control serum. The serum and antigen were mixed with a stirrer in circles. Mixing was done beginning with the 1:200 dilution. Twice, consecutively, the glass plate was rocked through four rotations, followed by incubation at 25±2 °C in an enclosed space for 4 min (±30 s). The white ceramic bioassay glass plate was rotated again and incubated for another 4 min (±30 s). The reaction was then read using a Minnesota box (NVSL, Ames, Iowa, United States).

### Statistical analysis

Data on the serological reactions were entered in Microsoft Office Excel 2010 (Microsoft Corp., Redmond, Washington, United States) and then exported to EPIINFO^TM^ 7.1.5.2 (Centers for Disease Control and Prevention, Atlanta, Georgia, United States) for analysis to determine proportions. The OpenEpi diagnostic test evaluation calculator^36^ was used to estimate parameters for the performance of the different screening tests compared to the RPPA test.

## Results

A total of 349 human residual blood samples were received at the clinical microbiology laboratory from the paediatric ward during the study period; 80 canine residual blood samples were received at the small animal veterinary clinic during the same time period. Of the 349 human samples, 105 were smear-negative for malaria; 244 were smear-positive and excluded from further analysis. Of the 105 malaria smear-negative samples, 44.8% (47/105) were from male patients and 55.2% (58/105) were from female patients. Seroprevalence of brucellosis among the malaria smear-negative residual blood samples was 23.8% (25/105) by Card, 23.8% (25/105) by BAPA and 23.8% (25/105) by SPT, but was 0% (0/105) using the RPPA test (Table 1). All the samples that tested positive using the Card test were also positive using the BAPA and the SP tests; therefore, there was a 100% agreement between Card, BAPA and SP tests. Of the 25 samples that tested positive using the three screening tests, none (0%, 0/25) were confirmed as positive using the RPPA test. Of the 25 human samples that were positive, 40% (10/25) were from male patients, and 60% (15/25) were from female patients. There was no significant difference between sero-reactions for male and female children (*p* = 0.131). The mean age of the seropositive children was 12 (±0.8) years.

**TABLE 1:** Sero-reactions to brucellosis tests for human and canine blood samples tested between January and June 2014 in Kampala, Uganda.

Sero-reaction	BAPA		Card		SPT		RPPA	
	*n*	%	*n*	%	*n*	%	*n*	%
**Humans (*n* = 105)**								
+	25	23.8	25	23.8	25	23.8	0	0.0
−	80	76.2	80	76.2	80	76.2	105	100.0
**Dogs (*n* = 80)**								
+	1	1.25	1	1.25	1	1.25	0	0.0
−	79	-	79	-	79	-	80	100.0

+/−, positive/negative; BAPA, buffered acidified plate antigen test; Card, brucellosis Card test; SPT, standard plate agglutination test; RPPA, rivanol precipitation plate agglutination test.

Eighty blood samples from dogs (one sample from each dog), all from households in the central region, were tested for *B. suis* antibodies, and only one was positive using the three screening serological screening tests – Card, BAPA and SPT. Thus, the seroprevalence of canine *B. suis* was 1.28% (1/80) using the screening tests. This sample was, however, negative using the RPPA test (seroprevalence using the RPPA test = 0%). There was no significant difference between the seropositivity for humans and dogs (*p* = 0.052).

Of the canine (1/80) and human (25/105) samples that tested positive by the SP test, all were positive at the two lowest dilutions (1:25 and 1:50). However, 60% (15/25) of the human samples tested positive at the third dilution (1:100) and 28% (7/25) tested positive at the fourth dilution (1:200).

For the human samples, the specificity of the screening tests (Card, BAPA and SPT) was 76.2% (67.2–83.3), sensitivity 0%, positive predictive value 0%, and the negative predictive value 100%. The Cohen’s Kappa statistic was 0.

For the canine samples, the specificity of the screening tests (Card, BAPA and SPT) was 76.2% (67.2–83.3), sensitivity 0%, positive predictive value 0%, and the negative predictive value 100%. The Cohen’s Kappa statistic was 0.

## Discussion

This study used three screening tests (Card, BAPA and SP) and an OIE confirmatory test (RPPA) to evaluate brucellosis as a possible aetiology of pyrexia of unknown origin among malaria smear-negative febrile children in a malaria-endemic African region. Using routine serological screening tests, the seroprevalence of brucellosis in dogs was 1.3%, and 23.8% in humans. There was 100% agreement between Card, BAPA and SP tests, but all were negative using the confirmatory RPPA test. The seroprevalence of brucellosis in both species using the OIE-recommended confirmatory serological test – RPPA test – was 0%.

Many serological studies conducted on brucellosis in endemic regions have reported a high prevalence of brucellosis.^37,38,39,40^ Unlike earlier studies, this study deployed an OIE confirmatory test in parallel with routinely used screening tests. The brucellosis prevalence reported without the use of a confirmatory test might be misleading, particularly when *Brucella* culture, the gold standard for *Brucella* spp. detection, is not carried out. *Brucella* culture is difficult to execute and suitable laboratory facilities are often lacking in resource-limited brucellosis-endemic regions. The discrepancy between the screening and confirmatory tests found in the current study is similar to that reported in a study conducted in Kenya.^41^

In this study, all the blood samples tested using the Card and other screening tests were negative when tested using the RPPA test. The difference in results can be explained by the fact that the most commonly used screening test in Uganda, the Card (Rose Bengal) test, deploys a *B. abortus/suis* antigen that would miss the detection of rough *Brucella* spp. such as *B. canis* and *B. ovis*.^42^ Also, cross-reaction is not uncommon when using Card (Rose Bengal), leading to false positives. The smooth lipopolysaccharide O-chain of smooth *B. abortus, B. melitensis* and *B. suis* and other Gram-negative bacteria expressing the lipopolysaccharide O-chain (such as *Escherichia coli* O157:H7 and *Yersinia enterocolitica* O:9) have been shown to cross-react with the Card (Rose Bengal) test (*B. abortus/suis* antigen).^43^ Also, the Card test does not differentiate between vaccinated and infected animals, or of the recovered individuals (as would be expected in a brucellosis-endemic setting) from those with active infection.^34^ On the other hand, the RPPA test is a quantitative and specific test which is able to rule out false-positive serological reactions.^34^ These factors could account for the difference in results between the Card and the RPPA test.

Additionally, even though there was agreement between the three serological screening tests (Card, BAPA and SP) the different screening serological tests target a different cluster of antibodies, some of which are not recognised by the OIE. For instance, the SP test lacks OIE recognition and detects immunoglobulin M, immunoglobulin G_2_, and immunoglobulin A (unpublished data, NVSL protocols, Ames Iowa, United States). While the BAPA test has OIE recognition, it detects mainly immunoglobulin G_1_ and immunoglobulin G_2_ and has a sensitivity ranging from 70% – 99%.^44^ Therefore, the use of any one of the screening tests in isolation could infer non-detection of some of the antibody types. In this study, the screening tests were in total agreement, meaning that at least one of the antibody types they target was present, pointing to possible prior exposure or other cross-reactions, but not infection (as this would be confirmed using the RPPA).

The RPPA test showed that all the test samples from humans and dogs were negative for brucellosis caused by the smooth *Brucella* strains. Specifically, the canine blood samples were negative for the highly pathogenic canine *B. suis*. The findings of this study contrasted with those of a similar study that showed a seroprevalence of 7.5% for human brucellosis in humans using the Card test alone.^3^ The difference could be attributable to the fact that they did not use a quantitative confirmatory serological test (RPPA) alongside the Card test.

Since a positive brucellosis test would necessitate long-term use of antibiotics,^45^ an incorrect brucellosis diagnosis (false-positive serological reaction) means that patients are placed on unnecessary antibiotics, this in turn aids the development of antibiotic resistance.^41^ Misdiagnosis of the causes of febrile illness in Uganda is a common cause of the misuse of antibiotics.^46^

### Limitations

The serological tests that were used in this study deployed antigens that could not aid the detection of rough colony *Brucella* spp. such as *B. canis* and *B. ovis. Brucella canis* is a species specific to dogs. Therefore, even though the residual samples were negative for *B. abortus* and *B. suis* they may not be negative for *B. canis*.

The Flourescent Polarisation assay is the recommended serological confirmatory test for brucellosis, but it was not used in this study; instead we used a supplemental confirmatory serological test. In this study we did not confirm infection by carrying out a culture for brucellosis.

To the best of our knowledge, the findings of this study can be used as per the scope of the study and in light of its limitations as clearly pointed out.

### Conclusion

This study used the traditional brucellosis screening tests (BAPA, Card and SP) in addition to a quantitative serological confirmatory test (RPPA). The additional use of RPPA confirmed as negative the positive results obtained using the BAPA, SP and Card tests. This implies that the improved specificity on using RPPA might help improve diagnostic accuracy by ruling out false-positive serological reactions. Since this study did not set out to detect *B. canis* in dogs, but rather canine *B. suis*, other serological tests specific to *B. canis* would help to complete the brucellosis picture in dogs.
